# Supplementary material for: The contribution of object identity and configuration to scene representation in convolutional neural networks
Source: PLoS One. 2022 Jun 28;17(6):e0270667. doi: 10.1371/journal.pone.0270667 (PMC9239439; doi:10.1371/journal.pone.0270667)
Supplement: Attachment — Submitted filename: ReplyLetter.pdf [file pone.0270667.s001.pdf]

We thank the reviewers for their time and effort, and their detailed and insightful comments on our manuscript. We have addressed in detail each comment raised by the reviewers below and have revised the manuscript accordingly. For easy referencing, we have included the reviewers' original comments in the italicized text below.

In the revised manuscript, we used track change to mark all the changes made to the manuscript. These include the revisions requested by the reviewers as well as some stylistic changes we made to further enhance the readability of the manuscript. We have replaced Marr (1980) with Marr (1982) as the latter was the intended reference. We have also reformatted the manuscript to meet PLOS ONE's style requirements.

****

3. Have the authors made all data underlying the findings in their manuscript fully available?

We have deposited all data underlying the findings as described above in a public repository (https://osf.io/7q5pj/). This information has been added to the cover page of the revised manuscript.

Reviewer #1:

The manuscript presents a thorough analysis of how different convolutional neural network (CNN) architectures respond to changes in object identity and configuration within a scene. The study appears methodologically sound. The choice of CNNs and variation in the scenes are mostly well-motivated and clearly described. What is unclear in the manuscript is the criteria for the selection of the objects within a scene and the relevance of spatial configuration of objects for scene processing at this level of analysis.

We thank the reviewer for the positive comments regarding our manuscript and for pointing out places where further improvements can be made. We have revised the manuscript thoroughly to address these concerns. Please see our detailed replies below to each comment.

- Previous studies on cortical scene processing emphasize the role of spatial layout of the scene (e.g., open vs. close, navigability of the scene), but what is the relevance of the exact configuration of the objects? A change in spatial configuration of objects likely has a significant effect for a familiar environment, but what is the role for a feedforward scene vision model?

We thank the reviewer for bringing up this point. Perceiving the exact configuration of objects in a scene is critical to how we interact with a scene. Although the spatial layout of a scene, as the reviewer described above, has been examined in prior research, to our knowledge, how object configuration is encoded during scene perception has not been studied. CNNs are trained for scene classification only. As such, the reconfiguration of the same set of objects into a new and reasonable arrangement does not change the nature of the scene (e.g., a bedroom scene is still a bedroom scene after the furniture is moved around), thus CNNs may not encode the precise object arrangement in a scene and could show a lack of sensitivity to such a configuration change. However, if objects and configurations are both fundamental elements of scene composition, then a system capable of classifying scenes should nevertheless explicitly encode these elements and demonstrate sensitivity to changes in both. Feedforward CNNs provide us with an excellent opportunity to test this idea.

We have added this discussion to p.3 of the revised manuscript. We believe clarifying this point has enabled us to better motivate the present study. We thank the reviewer for bringing this to our attention.

> *- Could the results also be interpreted as increased position-tolerant object processing across the layers? That is, the first layers are sensitive to configurational and even more to spatial shifts as they are sensitive to changes in low-level visual features whereas the end of the processing stream is more tolerant to such changes?*

We agree with the reviewer that position tolerance increases over the course of CNN visual processing. This can explain why towards the end of processing, object changes are represented more strongly than position and configuration changes. Nevertheless, even though configuration changes are smaller position changes in absolute magnitude than those of the uniform translation, by the end of CNN processing, we still observe greater sensitivity to a configuration change than to a translation. This shows that configurations are not represented in the same way as the absolute positions of the objects in a scene.

We have added this to the revised manuscript on p.28 to further clarify the difference between configuration and absolute position representation.

> *- More details would be needed about the selection of the objects. Also, the manuscript contains no details about how the scenes were modeled (software? automatic or hand-picked selection of objects?). Is the full set of scene images to be included with the final publication?*

We apologize for omitting these details in the manuscript. We chose to analyze indoor room scenes because room scenes are primary examples of real-world scenes with easily manipulable object/configuration information. In a bedroom scene for example, furniture can be rearranged or swapped without changing the categorization of the scene as a bedroom. CNNs have also been trained to classify different room scenes with high accuracy.

The fixed background, no background, and spatial shifted image collections were generated using Unreal Engine version 4.23 developed by Epic Games. We built the walls, floor, and ceiling of the room using static mesh objects with different materials. For example, rectangular wood objects in Unreal Engine comprise the floor layout of each scene. All images shown in these collections were taken from exactly the same perspective using the same lighting to showcase the 3D structure of the room and the furniture contained. All furniture was hand-picked from Unreal Engine's free object library to best represent typical objects in various indoor room scenes, e.g. couches in the living room. The variable background image collection was created using "us.mydeco.com" in 2011. This website appears to be no longer accessible. The full set of scene images are included in the data depository.

We have included the above information in the revised manuscript on p.9, and a discussion of why we chose to use indoor room scenes on p.7. We thank the reviewer for bringing this to our attention.

> *- How was the classification accuracy of the scenes defined? More details on what were considered as correct vs. incorrect classifications would be needed.*

We thank the reviewer for bringing this to our attention. We have included the requested information in the revised manuscript on pp.9-10. Specifically, we obtained each CNN's top five classification labels for a given image and assessed whether any of these labels would represent a reasonable classification of the scene. For example, in Figure 1A, the reasonable classifications of the image set were taken to be "dining room" and "living room" due to the presence of a large table and seating. If a reasonable classification was included among the CNN's top-5 classifications, we recorded a classification success, and if not, a failure. This was done separately for each of the four images in a set and the results were averaged over all images and sets to generate an average classification accuracy for each scene-trained CNN for a given image collection.

> *- The difference between classification accuracy and Euclidean distance results is intriguing. Why would the removal of the background have such drastic effect on classification accuracy but not on the other measures? Can you think this influencing how CNNs are compared with brain data?*

Because the CNN training set contains natural scene images with backgrounds present, CNNs expectedly learn that backgrounds are an essential part of a scene. It thus makes sense that when such backgrounds are removed, CNNs would no longer assign the same label to the scene. While human observers can disregard the presence of the background and see scenes without backgrounds as abstractions of the same scenes, CNNs are never trained to do so; we would assume that they can if such images are in the training set. Label assignment occurs at the very end of CNN scene processing. As such, up to this last stage, CNNs should be blind as to whether or not a scene would match one of its pre-learned labels. In this regard, CNN scene processing should not differentiate between scenes it can and cannot label. Similarly, humans may not be able to label a scene due to the presence of ambiguous information but this should not prevent their normal interaction with the scene otherwise.

We thank the reviewer for bringing up this discussion point. We have added the above discussion to the revised manuscript on p.26.

Reviewer #2:

In this study, the authors present computer generated images of 3D objects arranged in various scene configurations to assess the sensitivity of CNNs to changes in object configuration and object identity. Four different CNN architectures are assessed, and the CNNs were either pre-trained on a task of object classification (ImageNet) or scene classification (Places365). In general, the authors find greater sensitivity to object configuration than to object identity in the early layers of CNNs. This finding is to be expected given that changes in configuration likely lead to larger changes low-level image similarity, as some objects move to new previously unoccupied positions (from inspection of Fig 1). In the final layers for most CNNs, there appears to be greater sensitivity to object identity than to object configuration with the exception of DenseNet. It is claimed that there is a significant interaction effect between background type and layer but from inspection, it seems that the pattern of results are almost identical for no background and fixed background conditions. The authors should perform another analysis just comparing these two conditions because these two are visually the most comparable; the variable background condition introduces unintended shifts in viewpoint that make the interpretation of those results more problematic. From a glance, it appears that object background has little influence, and there does not seem to be any positive evidence to suggest the encoding of object-background relationships.

We have already included a direct ANOVA comparison between the fixed background collection and no background collection in the original manuscript (see p.18 of the

revised manuscript) and found a significant interaction between collection and layer. However, as we noted in the original manuscript these effects stemmed from minute numerical differences in the distance and index measures. The results from these two image collections otherwise exhibited an overall similar profile with the results being highly correlated over the course of CNN processing.

The authors report very similar results for the object-trained and scene-trained CNNs. While this is in some sense a negative finding, it is a somewhat surprising one. While the analyses and results presented in this paper do not provide a clear explanation for why the results turn out as they do, this finding is of scientific interest and provides potentially relevant information for future studies that wish to compare CNNs trained on objects vs. scenes. It raises the question as to what types of implicit knowledge / representations are these CNNs acquiring through their training, and how similar or different are these when the stimuli and tasks vary?

The reviewer raised a very interesting question here. This could be due to either the shared network architecture, a shared processing demand, or both. Due to shared network architecture, scene-trained and object-trained CNNs could utilize a common processing algorithm to perform both types of classification, with training providing the learning of the relevant input information for each task. Because both a scene and an object may contain configurations of more basic elements, i.e. a room scene with a particular arrangement of furniture and a complex object with a specific configuration of parts, the similar demand of scene and object classification may lead to the development of a convergent processing algorithm for both. We have added this discussion to the revised manuscript on pp.26-28.

The comparison of the object- and scene-trained CNNs with untrained CNNs provides positive evidence that sensitivity to object configuration and identity improves with training, and that the profiles observed across layers has some meaningful pattern to it, even though it is difficult to make strong conclusions about the observed pattern. The translation control analysis adds to this interpretation, as translation of a set of objects leads to stronger changes in the responses in the early layers but smaller changes in the near final layers.

In the discussion section, it would be helpful to provide more discussion about sensitivity to object configuration. Does this necessarily represent a genuine sensitivity to configuration or simply some form of implicit representation that is altered by shifts or relative shifts in object position? How might future studies further address or test this issue? The fact that the scene-trained CNNs performed very similarly to object-trained CNNs may suggest that both types of

CNNs simply encode objects, with the Place365 CNNs learning multiple objects that are predictive of particular scene categories without acquiring much sensitivity to relative object locations.

As the reviewer mentioned above, even though configuration changes are smaller position changes in absolute magnitude than those of the uniform spatial translation, by the end of CNN processing, we still observe greater sensitivity to a configuration change than to a translation. This shows that configurations are not represented in the same way as the absolute positions of the objects in a scene. That being said, it would be important for future studies to help us understand whether configuration is explicitly represented or whether it is implicitly coded among the objects in a scene. One manipulation could be to change the viewpoint of the scene. This would change some relative positions among the objects without changing the true configuration among the objects. Another manipulation could be to use a schematic representation of the configuration (e.g., by replacing all furniture in a room with cubes) and test its similarity with the actual room scene.

The fact that the scene classification performance drops when scene background is removed suggests that scene-trained CNNs do not only encode the objects in a scene, but also the texture and 3D structure provided by the presence of the background. See also our reply to the last comment regarding possible reasons why scene-trained and object-trained CNNs may produce similar results.

We have added some of the above discussion to the revised manuscript on pp.28-29

Overall, this study reports some interesting patterns and trends across layers regarding sensitivity to object configuration and object identity across layers for object- and scene-trained CNNs. While this study represents an initial foray into these questions, the information it provides will be useful for future investigations, and there will most certainly be many follow-up studies on these types of research questions. The analyses are clearly presented, generally well motivated, with some specific concerns noted (see below). The manuscript is clearly written and accessible. With further revision, I believe that this manuscript could be suitable for publication in PLOS One.

We thank the reviewer for the overall positive evaluation of the manuscript and for the comments raised. We hope our replies have satisfactorily addressed all of the reviewer’s concerns.

Specific comments

It is not clear whether the data shown in Figure 3 is drawn from object-trained CNNs, scene-trained CNNs, or both.

The data from Figure 2 is from scene-trained CNNs. We have added this information to the figure caption. We thank the reviewer for bringing this to our attention.

The use of the term kernel responses is not quite accurate when describing the fully connected layers, also kernels or filters do not respond (they are outputs), recommend rewording.... perhaps unit responses?

The reviewer is right that “unit responses” would be a more correct term to use. We have replaced “kernel responses” with “unit responses” throughout the revised manuscript. We thank the reviewer for this correction.

The use of z-normalized responses could potentially strongly affect the pattern of results, in particular the pattern of results observed across layers. It would be appropriate to perform the same set of analyses without normalization. Are the findings generally the same or quite different? Would be good to include either as supplementary figures or at least in the response letter to address these concerns.

We thank the reviewer for this comment. Attached below are z-normalized and non-z-normalized (original) object index measures for each of the network architectures for the fixed background collection. We observe very similar response profiles for both. Z-normalization removes amplitude differences among the images, layers and architectures and is a more conservative measure in our opinion.

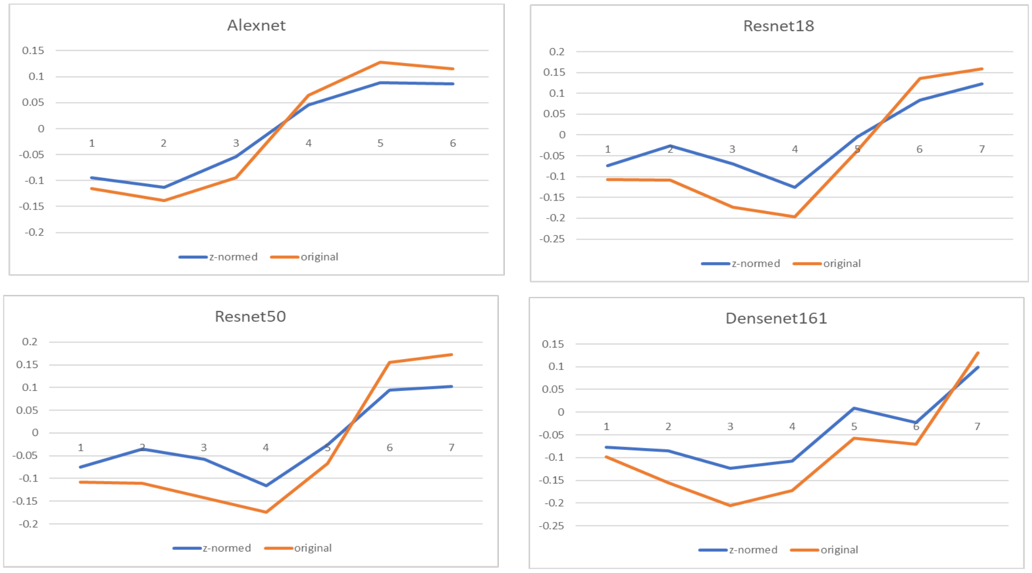

p. 16 - describing the trends across layers using correlation seems inappropriate. For example, if a very small change occurred across layers but this effect were highly consistent across CNN architectures, this would lead to a very strong positive correlation. Calculating the difference between last and first layers or reporting the slope of change would provide a more interpretable measure.

We report correlation between the index measure/distance measures and the rank order of the sampled layer throughout the paper as one method of quantifying the overall trajectory of the measure throughout the course of scene processing. The reviewer is correct in that a small change may lead to a strong correlation, but we do report the difference between the first and last layer where we report each correlation in the original manuscript (see Table 5). Overall, we believe that reporting correlation in addition to this difference gives additional characterization and thus presents a fuller, more representative picture than presenting either one measure alone.

"All untrained CNNs exhibit a monotonic decline in sensitivity to both types of changes as evidenced by strongly significant negative correlations of both Euclidean distance measures to the rank order of the sampled layer." - this wording is confusing

We have clarified above the text on p.22 of the revised manuscript. We apologize for this confusion.

In the manuscript, it is not clear how the data will be publicly shared.

We have deposited all data underlying the findings as described above in a public repository (https://osf.io/7q5pj/). This information has been added to the revised manuscript.

Reviewer #3:

This study reports a set of 'in silico' experiments in convolutional neural networks (CNNs), investigating how information about identity and configuration of objects embedded in computer-generated scene images is reflected in the internal activations of CNNs trained for object and scene recognition. The motivation to investigate these two dimensions (identity and configuration) separately and contrast them against one another is that both are thought to contribute to human scene processing, but that their relative importance is unclear. The motivation to study this process in CNNs is that previous work shows that internal activations in CNNs predict responses in scene-selective brain regions in humans. However, the 'computational algorithms' involved in scene processing in both CNNs and

brains remain unclear, and the authors suggest that investigating how CNNs process different aspects of a scene may shed light on these algorithms.

I reviewed this paper before, for a different journal. I was happy to see that relative to that previous submission, several comments were taken on board here. For example, the motivation for the study now comes across more clearly in the Introduction, the Results section is much more streamlined and readable and now includes important information about CNN accuracy. I see no major concerns with how the experiments were conducted or the statistical analyses and their outcomes. However still some issues remain, related to the logic behind the study, and if it indeed informs us about computational algorithms.

Major comments:

1. Inevitability of results in light of CNN architecture:

A major concern raised in the previous reviews was whether the results could be trivially related to the built-in architecture of the CNNs, which have a form of retinotopy (processing adjacent regions of visual space with adjacent kernels), whereby lower layers have higher spatial resolution compared to higher layers which process increasingly spatially pooled activations. Therefore, it doesn't seem very surprising that configuration information dominates in the earlier layers, and object information in later, fully connected layers that are more closely related to the task objective of labeling the image. I was surprised to see that this issue is not discussed in the current manuscript. I would very much like the authors to discuss their results in light of the architectural design of CNNs, i.e. to provide more theoretical embedding into how they expect the CNN to process these images, given their convolutional and pooling operations. Especially the comparison with untrained CNNs that is included in the current manuscript may speak to this issue, because untrained CNNs share the same architectural features with trained CNNs, but critically the training is lacking.

We appreciate the reviewer for bringing up this discussion point. Although we have touched up this in our discussion, we agree that a fuller treatment of this issue is needed. We have now expanded our discussion on this issue in the revised manuscript on p.28. Specifically, we do not believe that our main results from the trained CNNs could be accounted for entirely by the built-in CNN architecture as large response differences also existed between the trained and untrained CNNs sharing the same architecture. In Euclidean distance measures, instead of showing a uniform decline over the course of process as in the untrained CNNs, three out of the four scene-trained CNNs examined demonstrated more complex trajectories, and between consecutive

sampled layers, there was even an increase in the distance measures (see Figure 4b). This suggests that information about object and configuration changes is not only retained, but enriched in the mid layers of these trained CNNs. In object dominance index measures, in three out of the four CNNs, while the untrained CNNs showed an overall mild change over the course of processing with none of the indices reaching above zero by the end of processing, the scene-trained CNNs all showed much greater modulation with indices significantly above zero by the end of processing and significantly greater than those from the untrained CNNs (see Figure 4c). This shows that the dominance of configuration information in early layers and the dominance of object information in later layers is a result of CNNs being trained for the scene and object classification tasks. With such training, position tolerance increases over the course of CNN processing such that, towards the end of processing, object changes are represented more strongly than configuration changes. Nevertheless, even though configuration changes are smaller position changes in absolute magnitude than those of the uniform spatial translation, by the end of CNN processing, we still observe greater sensitivity to a configuration change than to a translation. This shows that configurations are not represented in the same way as the absolute positions of the objects in a scene.

2. Low accuracy of CNNs on these images:

As mentioned I think it's good the CNN accuracy is reported and its relation with the comparison of interest (configuration vs. object coding) investigated, but they also raise some questions:

** In the Methods: why only look at the scene-trained CNNs for this? How about the object trained CNNs? Do they classify the objects in the scenes correctly?*

We focused our initial classification analysis on the scene-trained CNNs as we used scene images in our study. These images contained small objects, different from the ones used to train CNNs for object classification. We thus anticipated that object classification accuracy could be poor from object-trained CNNs and our data indeed support that (the average object classification accuracy is now reported in Table 2). That being said, what is more critical here is not whether a CNN can match an object to a pre-learned label at the last stage of processing, but rather whether a CNN would show sensitivity to object changes throughout the course of CNN processing. Our results show that the latter was indeed the case.

** Methods, Page 7: 'manually assessed whether any of these labels represented a reasonable classification' – this sounds rather vague, can you give some concrete examples of what you considered reasonable in this context?*

We obtained each CNN's top five classification labels for a given image and assessed whether any of these labels would represent a reasonable classification of the scene. For example, in Figure 1A, the reasonable classifications of the image set were taken to be “dining room” and “living room” due to the presence of a large table and seating. If a reasonable classification was included among the CNN’s top-5 classifications, we recorded a classification success, and if not, a failure. This was done separately for each of the four images in a set and the results were averaged over all images and sets to generate an average classification accuracy for each scene-trained CNN for a given image collection. This has been added to the manuscript, and we thank the reviewer for bringing this up to our attention.

> ** Results: these are mainly focused on whether the classification accuracy impacts the relative representation of object vs. configuration information in the CNN (e.g. Page 13: ‘how a CNN represents object identity and configuration in a scene is not strongly affected by classification accuracy’). But I think the issue here is not necessarily how the accuracy impacts the representation, but more whether it’s meaningful and interesting to look at a CNN if it doesn’t really process these images correctly by any means? 50% accurate for top-5 is not exactly great performance. Why are CNNs then a relevant tool to study computational algorithms of scene processing, if they don’t perform well on the task?*

Compared to the real-world scene images used to train the CNNs, our computer-generated scene images contained overall less clues that would facilitate scene classification. For example, we only included four pieces of furniture in each room and we used a fixed room background. More furniture in a room and more distinctive room background would both aid scene classification. Additionally, in an attempt to create as many distinctive rooms as possible, some of the furniture included in a room may not uniquely label a room (e.g., a table could appear in either a living room or a dining room). This can explain why classification accuracy is low with our scene images.

It is important to note that classification label assignment occurs at the very end of CNN scene processing. As such, important low level processing may still occur within the CNN, despite its overall weakness at correctly supplying a high level classification of the scene. By analogy, humans may not be able to classify a scene due to the presence of insufficient or ambiguous information but this should not prevent their representation and interaction with the scene.

What is more critical here is whether a CNN would show sensitivity to object and configuration changes throughout the course of CNN processing. Our results show that the latter was indeed the case. Considering the Euclidean distance measures of Figure

5, our data demonstrates that imageNet trained CNNs increase their sensitivity to object and configuration changes; and more strikingly, these trained CNNs also enable this information to be propagated and enriched between subsequent layers of convolution. As CNNs are effectively a black box algorithmically, we cannot speculate as to the exact nature of processing in these CNNs, but our data shows that this scene relevant information is in fact being represented by the CNNs.

Indeed, as the literature has extensively show that CNNs are both exceptional at scene and object classification, and exhibit some similarity to the human brain, we assert that despite the overall weakness of CNNs at correctly classifying our images, they are nonetheless important visual processing systems and are, as our data shows, quite capable of processing and identifying the relevant scene information from our input stimulus set.

3. Equivalence of CNNs and brain region activations/representations

In a couple of places, some equivalences and comparisons between human/brain/fMRI and CNNs are made that I am uncomfortable with. In my opinion, such equivalences rely on assumptions that are not (yet) empirical grounded - not it this paper (which does not contain any human brain measures or behavior) nor previous literature. For example:

** Page 19: "Activate a scene-like representation in CNNs"; this statement assumes that CNNs have 'inner representations' that are scene-like, corresponding to our own introspective notion of seeing scenes as wholes (rather than collections of objects, for example); I'm not sure what such a representation would look like, all CNNs do is find relevant features in images to correctly match it with a prescribed label, whether those features are scene-like or not seems is not (and perhaps cannot be) clearly defined.*

The reviewer has a good point here. We have taken this language out of the revised manuscript on p.28.

** Page 19: "Given that both scene-trained and object-trained CNNs exhibited similar responses in the present study, it remains possible that scene processing in CNNs could be more closely related to the LOC than scene processing in the human brain"; this statement assumes that a scene-trained CNN 'should be' more like a scene-processing brain region and an object-trained CNN more like a LOC region. As far as I know it is as of yet unclear whether we can think of these regions as being more or less CNN like (many CNNs seem to have some correlation with all ventral region activity), and to me this prediction reads like an*

unfounded form of reverse inference from CNNs to brains (I also don't quite follow why it should go in the direction of LOC: couldn't it equally well suggest that object processing in CCNs is more related to how PPA processes objects?)

We agree with the reviewer here that there are a fair number of unknowns and things could go either way. In fact, King et al. (2019, Neuroimage) showed that the middle layer of an ImageNet training VGG-S network showed the highest correlation with both LOC and PPA. We have revised our discussion on this point in the revised manuscript on p.29. Specifically, we now include the following: "While scene processing differences clearly exist in the human brain between object and scene-processing regions, both scene-trained and object-trained CNNs exhibited similar responses in the present study. This is consistent with the finding of King et al. (2019) who also showed that the middle layer of an ImageNet trained VGG-S network showed the highest correlation with both LOC and PPA. It would be interesting in future studies to present human participants the images used here and measure their fMRI responses in both object- and scene-selective regions of their brains and examine whether a similar response profile is present in both regions of the human brain. Doing so would not only enrich our understanding of object and scene processing in the human brain, but also identify potential processing differences between the human brain and CNNs".

Minor comments:

Abstract:

The conclusion is not really a summary of the finding, but rather a description of the study.

We have revised the abstract to include a more precise summary of the findings. We thank the reviewer for this comment.

Intro:

** Page 3: Not only Groen et al., showed correspondence between CNN features and fMRI responses in scene regions in the brain, but also Bonner & Epstein 2017 (PNAS), Dwivedi 2020 (JOCN) King et al. 2019, (NeuroImage), to name a few.*

We thank the reviewer for bringing these additional studies to our attention. We believe the reviewer perhaps had in mind Bonner & Epstein (2018, PLOS Computational Biology) and not Bonner & Epstein (2017, PNAS). We have added these studies to the revised manuscript on p.3.

** Page 3: 'How these two types of information are weighted during scene processing has not been systematically examined in either the human brain or CNNs'. That's quite a statement to make – especially for CNNs, there are so many publications on deep learning across many conferences and journals… So maybe better to say 'to our knowledge, this has not been examined….'?*

The reviewer is correct that our statement was too strong. We have revised the text according to the reviewer's suggestion.

** Page 4: How is similarity between brain and CNN 'utilized' in this study? Do you perhaps mean 'motivated by'?*

We agree with the reviewer's suggestion here and have revised the text accordingly.

Methods:

** Page 5: the motivation for CNN selection still very brief, a bit more background (e.g. what are residual blocks in ResNets and why are they 'novel'?) would be helpful. A phrase like 'were chosen for their convolution depth' is not really informative; why is convolution depth interesting/important to consider when investigating scene processing?*

We have added more motivations for CNN selection. Regarding ResNet, residual networks trained for object classification are considered to be the first to surpass human performance, with residual modules helping the gradient flow by allowing it to bypass processing layers that would cause it to dissipate and disrupt learning and enabling the model to learn appropriate processing depth for a task (Serre, 2019). We considered varying convolution depth important as it is a prominent network feature and may differentiate performance from the different networks. Together, the networks tested here provided a diverse network architecture with varying depth, complexity, and degree of interconnectivity. It worth noting that previous brain and CNN studies have also included some of these networks, with Alexnet examined in Bonner & Epstein (2018), Groen et al. (2018) and Dwivedi et al. (2020), and Resnet18 and Resnet50 examined in Dwivedi et al. (2020). We have added this information to the revised manuscript on p.5.

** Page 5: 'inside the convolution' – awkward phrasing, 'in the hierarchy' would be more appropriate, I think.*

We meant inside the network hierarchy. We have clarified this in the revised manuscript.

** Page 7: 'reversal of magnitude' – magnitude of what? Unit activations?*

It is the reversal of response magnitude in the index measures. We have clarified this in the revised manuscript.

Results:

** Page 11: 'All statistical tests performed used image set as dependent variable' – this seems inaccurate to me, the dependent variable is Euclidean distance. Do you mean that the image sets are the individual data points going into the statistical test (and that the error measures reflect the error across image sets)?*

The reviewer is correct here. What we meant was that the Euclidean distances from each image set were the dependent variables (i.e., the image sets provided the individual data points for the statistical tests with the error measures reflecting the error across the image sets). We have corrected this in the revised manuscript.

** Page 11: 'near significant' is not a thing… you can talk about a trend perhaps.*

We have now replaced the term with "marginally significant", which is a more standard term.

** What are the units in Table 2? Percent (0-100), or a score between 0 and 1?*

It is a score between 0 and 1, expressing the proportion of successful classifications. We have clarified this in the revised manuscript.

Discussion:

** Page 18: "But also can shed significant insights on the likely computational algorithms used by the human brain during scene processing". I would be careful claiming this, because a) human scene processing is not investigated in this study and b) we don't know if insights acquired by doing so would indeed be 'significant'.*

We have revised our wording to avoid making a strong claim. We now state that "studying the details of scene processing in CNNs not only can enrich our understanding of the computational algorithms governing scene processing in CNNs, but also provide guidance as to how CNNs may further be used to study and understand scene processing in the human brain".

** Page 19: "Removing spatial background had only minor effects on the sensitivity of a CNN on object or configuration changes" – but it had a big effect on accuracy, isn't that more relevant when considering activity in PPA?*

As stated in earlier replies, there are a number of factors that could contribute to the low CNN classification accuracy. Consequently, failure to classify a scene may not necessarily lead to a lower PPA activity. Past studies have not measured PPA responses to the no-background scenes used in the present study. Whether PPA would indeed show a differential response to such scenes awaits further research.
